# The impact of regional block presence on large language model–based postoperative analgesia recommendations in abdominal surgery: a comparative study using real-world patient data

**DOI:** 10.1186/s12871-026-03814-y

**Published:** 2026-04-10

**Authors:** Bahar Uslu Bayhan, Tuğçe Gazioğlu Kişi

**Affiliations:** Department of Anesthesiology and Reanimation, Gaziantep City Hospital, Gaziantep, Türkiye

**Keywords:** Postoperative analgesia, Regional anesthesia, Multimodal analgesia, Artificial intelligence, Large language models

## Abstract

**Background:**

Postoperative pain management is a core component of anesthesiology practice, with regional anesthesia playing a key role in multimodal analgesia strategies. Large language model (LLM)–based artificial intelligence (AI) systems are increasingly proposed as clinical decision support tools; however, their ability to integrate critical perioperative context, such as the presence of an existing regional block, remains insufficiently explored.

**Methods:**

This prospective, observational, comparative study included 144 adult patients undergoing elective abdominal surgery at a tertiary care center, after exclusion of four patients due to severe preoperative or intraoperative complications that significantly altered the planned postoperative analgesia. Patients were grouped according to the presence or absence of a regional block (70 per group). For each patient, anonymized and standardized clinical scenarios were evaluated independently by three LLM-based AI systems (ChatGPT, Gemini, and Copilot) to generate postoperative analgesia recommendations. AI outputs were assessed by blinded anesthesiology experts for opioid recommendation, multimodal analgesia, consideration of regional anesthesia, and overall clinical appropriateness using a 5-point Likert scale. Multivariable logistic and ordinal logistic regression analyses were performed to determine the independent effect of regional block presence, adjusting for relevant clinical covariates. Agreement between AI recommendations and actual clinical practice was evaluated using Cohen’s kappa.

**Results:**

Regional block presence was not independently associated with opioid recommendations generated by any AI system (all *p* > 0.05). However, the likelihood of recommending an additional regional block was significantly reduced by ChatGPT (adjusted odds ratio [aOR] 0.02, *p* < 0.001) and Copilot (aOR 0.15, *p* = 0.019). Gemini demonstrated complete separation, consistently recommending regional blocks only in patients without an existing block. Multimodal analgesia was universally recommended by ChatGPT and Gemini, precluding regression analysis. Expert evaluation scores were significantly higher in scenarios with an existing regional block across all AI systems. Overall agreement between AI-generated recommendations and real-world clinical decisions was limited.

**Conclusions:**

LLM-based AI systems demonstrate partial contextual awareness of regional anesthesia when generating postoperative analgesia recommendations. However, this awareness does not consistently translate into concordance with real-world clinical practice. These findings support the use of AI as an adjunctive decision support tool rather than a substitute for clinician judgment in postoperative pain management.

**Supplementary Information:**

The online version contains supplementary material available at 10.1186/s12871-026-03814-y.

## Introduction

Artificial intelligence (AI) has assumed an increasingly prominent role in pattern recognition, prediction, and decision-support processes in recent years, driven by rapid advances in computational power and data-processing capacity. In the healthcare domain, AI-based applications offer substantial opportunities for synthesizing clinical data, risk stratification, and the development of clinical decision support systems. Nevertheless, particularly in clinical decision-making processes that directly affect patient safety, the reliability, contextual awareness, and potential risks of AI-generated recommendations remain subjects of ongoing debate [1, 2].

The concept of artificial intelligence was first introduced by John McCarthy in 1956 and has since evolved from rule-based systems toward data-driven and learning-based models. Among the most recent developments in this evolution are large language models (LLMs), which have attracted growing interest in medicine due to their ability to interpret natural language inputs and generate context-sensitive responses [2–4]. Given the inherent complexity of clinical decision-making, interindividual variability, and strong dependence on clinical context, the integration of AI into medical practice necessitates careful and systematic evaluation.

In perioperative medicine and anesthesiology, AI-based decision support systems have been investigated across a range of applications, including preoperative risk assessment, prediction of intraoperative hemodynamic instability, airway management, estimation of postoperative complications, and optimization of pain management strategies [1, 5, 6]. These systems are generally positioned not as autonomous decision-makers but as supportive tools designed to assist clinicians, with the responsibility for final clinical decisions remaining firmly with the physician.

Postoperative pain management is a fundamental component of anesthesiology practice, exerting a substantial influence on patient comfort, recovery trajectories, and complication rates. Contemporary guidelines advocate multimodal analgesia strategies to minimize opioid-related adverse effects and emphasize the central role of regional anesthesia techniques within this framework [7–9]. In patients undergoing abdominal surgery, regional block techniques have been shown to reduce opioid requirements and facilitate more effective and safer postoperative pain control [10]. Despite this, it remains unclear to what extent AI-based systems incorporate critical clinical context variables—such as the presence of a regional block—when generating postoperative analgesia recommendations. The existing literature has largely focused on the accuracy or consistency of AI-generated medical advice, while studies examining how such recommendations vary according to specific perioperative interventions remain limited [2, 11].

Therefore, the primary aim of this study was to evaluate, using real-world patient data, the impact of regional block presence on postoperative analgesia recommendations generated by contemporary AI systems in patients undergoing abdominal surgery. Additionally, we sought to assess the clinical appropriateness of these recommendations and to determine the extent to which AI systems successfully integrate regional anesthesia—a key perioperative variable—into postoperative analgesia planning.

## Methods

### Study design and ethical approval

This study was designed as a prospective, observational, and comparative investigation conducted at the Department of Anesthesiology and Reanimation of Gaziantep City Hospital. The study protocol was approved by the Gaziantep City Hospital Non-Interventional Clinical Research Ethics Committee (approval decision no. 312/2025; approval date: 17 September 2025). The research was carried out in accordance with the principles of the Declaration of Helsinki and relevant national regulations.

Written informed consent was obtained from all participants prior to enrollment. Patient confidentiality was strictly maintained throughout the study, and all data were anonymized before analysis.

### Inclusion criteria

Patients who met all of the following criteria were included in the study:

- Age ≥ 18 years.
- Undergoing elective abdominal surgery (open or laparoscopic).
- Surgery performed under general anesthesia or regional anesthesia.
- Availability of complete perioperative clinical data.
- A planned postoperative pain management strategy using systemic analgesics, regardless of whether regional anesthesia was applied postoperatively.

### Exclusion criteria

Patients were excluded if they met any of the following criteria:

- Age < 18 years.
- Inability to provide informed consent due to mental or neurological conditions.
- Undergoing non-abdominal surgical procedures.
- Surgery performed exclusively under local anesthesia or sedation.
- Development of severe preoperative or intraoperative complications that significantly altered the planned postoperative analgesia.
- Presence of incomplete or inconsistent perioperative data.

In addition, patients whose postoperative analgesia plans were substantially modified due to unexpected postoperative complications or the need for intensive care unit admission were excluded from the final analysis.

### Data collection

Perioperative data were collected prospectively from patient medical histories, anesthesia records, and the hospital electronic medical record system. Demographic variables included age, sex, and body mass index (BMI). Clinical variables comprised the American Society of Anesthesiologists (ASA) physical status classification, the presence and type of comorbidities, and the surgical approach (open or laparoscopic).

Anesthesia-related variables included the type of anesthesia administered, whether regional anesthesia was applied, and the specific type of regional block performed, such as transversus abdominis plane (TAP) block, bilateral TAP block, quadratus lumborum block, or erector spinae plane block. Postoperative analgesia variables included the use of opioid and non-opioid analgesics, the type of opioid administered, total opioid dose, and whether a multimodal analgesia strategy was implemented.

Although the core literature review guiding the study design was completed prior to ethical approval, additional references published after the approval date were incorporated during manuscript preparation solely to contextualize the findings and reflect recent developments in the field, without influencing the study design, data collection, or analysis.

### Generation of AI-based clinical scenarios

For each patient, anonymized and standardized clinical scenarios were generated using the collected perioperative data. Patients were categorized into two groups based on the presence or absence of a regional block (with block vs. without block). To ensure consistency across all artificial intelligence systems, the clinical scenarios were prepared in a structured and uniform format.

### Artificial intelligence systems and query process

The generated clinical scenarios were independently evaluated using three large language model–based artificial intelligence systems (ChatGPT, Gemini, and Copilot). For each patient, postoperative analgesia recommendations were obtained separately from each AI system, and the raw text outputs generated by the models were recorded verbatim.

All AI queries were conducted in English. Each clinical scenario was queried once per AI system to reflect a realistic single-use clinical decision-support setting; repeated querying was intentionally avoided to prevent stochastic variability.

No editing, prompting adjustments, or clinical intervention was applied to the AI outputs. The recommendations generated by the AI systems were not used for clinical decision-making and were evaluated solely for observational and comparative research purposes.

AI-generated postoperative analgesia recommendations were obtained using three publicly available large language model–based systems: ChatGPT (OpenAI), Gemini (Google), and Copilot (Microsoft). All queries were performed between 1 October and 31 October 2025. During this period, ChatGPT was accessed via the web interface using the GPT-4–class model as reported by the platform at the time, Gemini via the Gemini web interface (current publicly available version), and Copilot via the Microsoft Copilot web interface.

All AI systems were queried within the same predefined time frame using an identical prompt template. No prompt tuning, follow-up queries, or model setting adjustments were applied. To the best of our knowledge, no major publicly announced model updates affecting these systems occurred during the query period. The proprietary nature of large language models limits access to detailed internal versioning; however, all analyses reflect the model behavior as available to end users at the time of data collection.

All AI systems were queried using an identical, predefined prompt template incorporating patient demographics, surgical characteristics, and anesthesia-related variables derived from real-world clinical data. The prompt was applied uniformly across all cases without modification, prompt tuning, or follow-up queries. The complete prompt template is provided in Supplementary Appendix 1.

### Evaluation of AI-generated analgesia recommendations

Postoperative analgesia recommendations generated by the artificial intelligence systems were evaluated using two complementary approaches.

First, agreement analyses were performed by comparing AI-generated recommendations with the actual postoperative analgesia strategies implemented in routine clinical practice, as documented in patient medical records. These real-world clinical decisions served as the reference standard exclusively for agreement analyses.

Second, the clinical appropriateness of AI-generated recommendations was independently assessed by two anesthesiology specialists with at least three years of clinical experience. The evaluators were blinded to both the identity of the AI system and to each other’s assessments. In cases of disagreement, consensus was achieved through consultation with a third senior anesthesiology specialist.

AI-generated free-text outputs were translated into structured variables using predefined criteria. Two anesthesiology specialists independently coded each recommendation for opioid use, multimodal analgesia, and regional anesthesia as binary outcomes (yes/no). Explicit recommendations were coded as “yes,” whereas the absence of a recommendation was coded as “no.” Overall clinical appropriateness was rated using a 5-point Likert scale ranging from 1 (completely inappropriate) to 5 (fully appropriate). Discrepancies in coding or scoring were resolved by consensus with a third senior anesthesiologist.

Expert evaluations were conducted independently of real-world clinical decisions and were not used as the reference standard in agreement analyses.

### Outcomes

The prespecified primary outcome of the study was the AI-generated recommendation regarding postoperative opioid use (yes/no). This outcome was selected a priori because opioid-related decision-making represents a clinically critical and actionable component of postoperative analgesia planning.

Secondary outcomes included AI-generated recommendations for multimodal analgesia (yes/no), inclusion of regional anesthesia in the postoperative analgesia plan (yes/no), and overall clinical appropriateness of AI-generated recommendations as assessed by expert anesthesiologists using a 5-point Likert scale.

### Statistical analysis

Continuous variables are presented as mean ± standard deviation or median (interquartile range), depending on data distribution, while categorical variables are reported as counts and percentages. Between-group comparisons were performed using the independent samples *t*-test or the Mann–Whitney *U* test for continuous variables, and the chi-square test or Fisher’s exact test for categorical variables, as appropriate.

To evaluate the independent effect of regional block presence on AI-generated postoperative analgesia recommendations, multivariable logistic regression analyses were conducted. Ordinal logistic regression models were used to analyze expert evaluation scores. All regression models were adjusted for age, sex, body mass index (BMI), American Society of Anesthesiologists (ASA) physical status classification, and surgical approach (open vs. laparoscopic), with surgical approach included to account for the observed between-group imbalance. Regression analyses were performed separately for each AI system (ChatGPT, Gemini, and Copilot). Data were not pooled across AI systems; therefore, no adjustment for within-patient clustering was required.

For sensitivity analyses, expert-rated Likert scores were collapsed into broader ordinal categories (1–2 = low, 3 = moderate, 4–5 = high appropriateness) to reduce sparsity and assess the robustness of ordinal regression estimates.

In outcomes exhibiting complete separation, penalized logistic regression with bootstrap confidence intervals was applied. Agreement between AI-generated recommendations and expert clinical decisions was assessed using percentage agreement, Cohen’s kappa coefficient, sensitivity, and specificity.

A two-sided *p* value of < 0.05 was considered statistically significant for all analyses.

### Sample size calculation

The sample size calculation was performed using G*Power software (version 3.1.9.7) during the study planning phase. In the absence of prior data allowing reliable a priori estimation of odds ratios for AI-generated postoperative analgesia recommendations, the calculation was initially based on detecting a moderate between-group difference, with an alpha error probability of 0.05 and a statistical power of 80%. Based on this approach, a minimum of 64 patients per group was required. To account for a potential data loss rate of approximately 10%, it was planned to include at least 140 patients in total.

Given that the primary outcomes of the study were binary or ordinal and were ultimately analyzed using logistic and ordinal logistic regression models, the adequacy of the final sample size was additionally assessed using established recommendations for multivariable regression analyses, including events-per-variable considerations. With a total of 140 patients and a limited number of covariates included in the adjusted models (age, sex, body mass index, ASA physical status, and surgical approach), the sample size was considered sufficient to support stable and reliable regression estimates for the planned analyses.

## Results

**Fig. 1:**
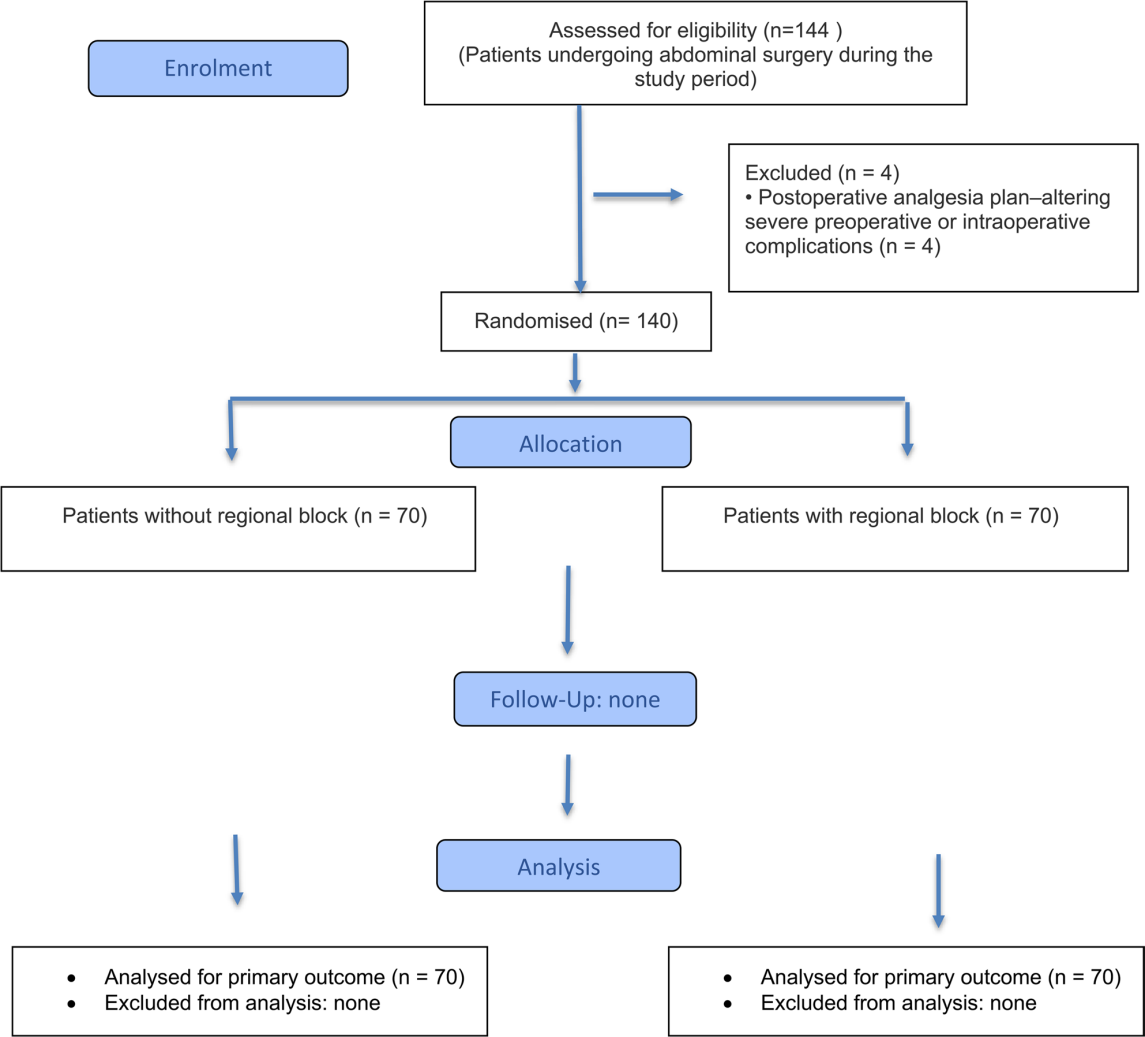
CONSORT-style flow diagram of patient enrolment and analysis

The patient flow diagram is presented in (Fig. 1). A total of 140 patients undergoing abdominal surgery were included in the analysis, with 70 patients in the regional block group and 70 in the no-block group. Baseline demographic and clinical characteristics, including age, body mass index, and ASA physical status, were comparable between groups (all *p* > 0.05). (Table 1)The regional block group included a significantly higher proportion of open surgical procedures compared with the no-block group (*p* = 0.027).

**Table 1:** Demographic and clinical characteristics of patients according to regional block status

Variable	No regional block (*n* = 70)	Regional block (*n* = 70)	*p* value
Age (years)	52.0 ± 19.0	48.8 ± 18.8	0.33
Female sex, *n* (%)	44 (62.9%)	40 (57.1%)	1.00
BMI (kg/m²)	29.3 (23.9–32.8)	29.6 (25.1–33.1)	0.37
ASA score, median (IQR)	2 (2–3)	2 (2–3)	0.81
Smoking, *n* (%)	18 (25.7%)	12 (17.1%)	0.30
Alcohol use, *n* (%)	14 (20.0%)	10 (14.3%)	0.50
Surgical approach			0.027
• Open surgery, *n* (%)	31 (44.3%)	45 (64.3%)	
• Laparoscopic surgery, *n* (%)	39 (55.7%)	25 (35.7%)	

Values are expressed as mean ± SD, median (IQR), or *n* (%). Group comparisons were performed using appropriate parametric or nonparametric tests. A *p* value <0.05 was considered statistically significant

*Abbreviations*: *ASA* American Society of Anesthesiologists physical status classification, *BMI* body mass index, *SD* standard deviation, *IQR* interquartile range

The adjusted effects of regional block on AI-generated postoperative analgesia recommendations are summarized in Table 2.

**Table 2:** Adjusted effect of regional block on AI-generated postoperative analgesia recommendations and expert evaluation

AI system	Outcome	Adjusted effect of regional block (aOR, 95% CI)	*p* value	Method
ChatGPT	Opioid recommended by AI (yes)	0.57 (0.26–1.24)	0.155	Logistic regression
ChatGPT	Regional block recommended by AI (yes)	0.02 (0.00–0.18)	< 0.001	Logistic regression
Gemini	Opioid recommended by AI (yes)	1.13 (0.46–2.79)	0.792	Logistic regression
Gemini	Regional block recommended by AI (yes)	0.00 (0.00–0.00)*	NA	Penalized logit (bootstrap CI)
Copilot	Opioid recommended by AI (yes)	0.54 (0.25–1.17)	0.117	Logistic regression
Copilot	Multimodal analgesia recommended by AI (yes)	6.26 (0.71–55.15)	0.098	Logistic regression
Copilot	Regional block recommended by AI (yes)	0.15 (0.03–0.73)	0.019	Logistic regression
ChatGPT	Higher expert evaluation score (ordinal, 1–5)	182.28 (31.09–1068.72)	7.99e-09	Ordinal logistic regression
Gemini	Higher expert evaluation score (ordinal, 1–5)	4.42 (1.05–18.61)	0.043	Ordinal logistic regression
Copilot	Higher expert evaluation score (ordinal, 1–5)	481.16 (62.40–3710.42)	3.10e-09	Ordinal logistic regression

Adjusted models included age, sex, body mass index, ASA physical status, and surgical approach

Odds ratios represent the effect of regional block presence

*Abbreviations*: *aOR* adjusted odds ratio, *CI* confidence interval, *ASA* American Society of Anesthesiologists physical status classification

* Complete separation observed; penalized logistic regression with bootstrap confidence intervals was applied

Extreme odds ratios with wide confidence intervals observed in certain models were attributable to sparse data and complete separation and should be interpreted as reflecting deterministic model behavior rather than precise effect size estimates.

After adjustment for age, sex, BMI, ASA score, and surgical approach (open vs. laparoscopic), the presence of a regional block was not independently associated with opioid recommendation by ChatGPT (aOR 0.57, 95% CI 0.26–1.24; *p* = 0.155), Gemini (aOR 1.13, 95% CI 0.46–2.79; *p* = 0.792), or Copilot (aOR 0.54, 95% CI 0.25–1.17; *p* = 0.117).

In contrast, regional block presence had a marked impact on AI block recommendations. ChatGPT was significantly less likely to recommend an additional regional block when a block had already been performed (aOR 0.02, 95% CI 0.00–0.18; *p* < 0.001). Similarly, Copilot showed a significantly reduced likelihood of recommending a block in the presence of an existing regional block (aOR 0.15, 95% CI 0.03–0.73; *p* = 0.019).

For Gemini, complete separation was observed: the model recommended a regional block for all patients without a block, whereas it rarely did so when a block was already present. Due to lack of outcome variability, classical logistic regression could not be fitted; therefore, penalized regression with bootstrap confidence intervals was applied.

Multimodal analgesia recommendations could not be modeled for ChatGPT and Gemini, as both systems recommended multimodal analgesia in 100% of cases, resulting in no outcome variability. (Tables 3, 4 and 5).

**Table 3:** Agreement between AI-generated postoperative analgesia recommendations and expert clinical decisions. A. Agreement for opioid use

AI system	Agreement (%)	Cohen’s κ	Sensitivity	Specificity
ChatGPT	55.7	−0.01	0.33	0.56
Gemini	52.9	−0.02	0.39	0.55
Copilot	58.6	0.07	0.41	0.60

**Table 4:** Agreement between AI-generated postoperative analgesia recommendations and expert clinical decisions. B. Agreement for multimodal analgesia

AI system	Agreement (%)	Cohen’s κ	Sensitivity	Specificity
ChatGPT	82.1	0.00	1.00	0.00
Gemini	82.1	0.00	1.00	0.00
Copilot	82.9	0.19	0.97	0.16

**Table 5:** Agreement between AI-generated postoperative analgesia recommendations and expert clinical decisions. C. Agreement for regional block recommendation

AI system	Agreement (%)	Cohen’s κ	Sensitivity	Specificity
ChatGPT	85.7	−0.03	0.00	0.87
Gemini	83.6	NA*	1.00	0.00
Copilot	90.0	−0.03	0.00	0.91

Expert clinical decisions were derived from actual postoperative analgesia records and used as the reference standard

Agreement was assessed using percentage agreement and Cohen’s kappa (κ). Sensitivity and specificity were calculated with expert clinical decisions as the reference

*Abbreviations*: *κ* Cohend's kappa,* NA *not applicable

* Complete separation observed, precluding calculation of κ

High crude agreement accompanied by low or near-zero Cohen’s kappa values—particularly for multimodal analgesia and regional block recommendations—reflects highly imbalanced recommendation patterns and the well-described prevalence effect associated with kappa statistics. Agreement between AI-generated recommendations and real-world clinical practice varied across analgesic domains: concordance for opioid use was low across all AI systems, with kappa values close to zero or negative, whereas multimodal analgesia demonstrated high raw agreement but low specificity due to near-uniform recommendations. For regional block recommendations, overall agreement rates were high, yet kappa values remained low, indicating systematic recommendation behaviors rather than true decision-level concordance. Complete separation was observed for Gemini in regional block recommendations, precluding calculation of Cohen’s kappa.

## Discussion

Postoperative pain management is as critical as the surgical procedure itself, as it directly influences patient comfort, recovery, and overall outcomes. The existing literature provides strong support for multimodal analgesia strategies and regional anesthesia techniques in reducing opioid requirements in the postoperative period [7, 12]. For instance, clinical guidelines recommend multimodal analgesia to minimize opioid use while optimizing pain control, and regional block techniques have been shown to enhance analgesic efficacy and reduce opioid consumption [13, 14]. In this context, the present study represents one of the first comparative investigations to evaluate, based on real-world patient data and routine clinical practice, the impact of regional anesthesia on postoperative analgesia recommendations generated by large language model–based artificial intelligence systems in patients undergoing elective abdominal surgery.

Our findings demonstrate that the presence of a regional block did not independently influence opioid recommendations generated by ChatGPT, Gemini, or Copilot. Previous studies in the field of AI-driven perioperative pain management have primarily focused on predicting postoperative pain outcomes or persistent opioid use using multivariable clinical datasets and composite risk factors. Consequently, the automatic translation of an intervention-specific variable—such as regional block application—into opioid-related recommendations may not be consistently expected [15]. In our multivariable analyses, the lack of a significant change in opioid recommendation patterns despite the presence of a regional block suggests that AI systems may prioritize surgical characteristics, patient-related factors, or generalized pain management principles over intervention-specific analgesic strategies. The higher prevalence of open surgery in the regional block group likely reflects real-world clinical decision-making, whereby regional anesthesia techniques are more frequently employed in patients undergoing more invasive procedures with greater anticipated postoperative pain. Importantly, surgical approach was accounted for in all adjusted analyses, and the observed associations between regional block presence and AI-generated recommendations remained robust after controlling for this potential confounder.

This observation indicates that current AI systems do not inherently adopt a regional anesthesia–centered, opioid-sparing approach when formulating postoperative analgesia recommendations [8, 16]. Furthermore, the reliance of AI-generated outputs on correlation-based pattern recognition, coupled with potential inconsistencies in capturing nuanced clinical guidelines and contextual details, may contribute to the inconsistent prioritization of opioid-sparing strategies across individual cases [1, 17].

In contrast, the presence of a regional block exerted a pronounced effect on the propensity of AI systems to recommend regional anesthesia. Both ChatGPT and Copilot showed a significant reduction in the likelihood of suggesting an additional regional block in patients who had already received one, whereas complete separation was observed for the Gemini system. Specifically, Gemini consistently recommended regional blocks in all patients without an existing block, while nearly completely eliminating such recommendations in patients who had already undergone regional anesthesia. Previous studies have suggested that AI systems are capable of treating distinct clinical interventions—such as regional anesthesia—as strong contextual determinants and may integrate such information more rigidly into their decision-making processes [2, 18, 19]. This finding implies that Gemini, in particular, may interpret the presence of regional anesthesia as a dominant contextual cue and incorporate it into its recommendations in a more deterministic manner [2, 18].

When multimodal analgesia recommendations were examined, no statistical variability was observed for ChatGPT and Gemini, as both systems uniformly recommended a multimodal approach across all cases. This pattern suggests that these AI systems treat multimodal analgesia as a universally endorsed standard emphasized in contemporary guidelines, with limited sensitivity to patient- or surgery-specific differentiation. Consistent with this interpretation, the literature indicates that AI-based decision support systems in pain management often provide guideline-driven and generalized recommendations, while reflecting patient-specific nuances to a limited extent [1, 15, 20]. Although Copilot demonstrated greater variability in multimodal analgesia recommendations, this difference did not reach statistical significance.

With respect to expert evaluation scores, the presence of a regional block was associated with higher overall clinical appropriateness ratings across all AI systems. Prior reports have shown that AI-generated recommendations are perceived as more acceptable by clinicians when clinical scenarios more closely reflect relevant contextual information [21–23]. However, expert evaluation scores primarily reflect the perceived clinical appropriateness of AI outputs and may not necessarily indicate concordance with actual clinical practice. Accordingly, it has been recommended that expert-based assessments be complemented by objective agreement analyses when evaluating AI-generated clinical recommendations.

In line with this approach, the agreement analyses performed in the present study revealed a limited relationship between AI-generated recommendations and expert clinical decisions. Cohen’s kappa values calculated for opioid use were close to zero or negative across all systems, indicating poor concordance between AI recommendations and real-world clinical practice. Although high crude agreement rates were observed for multimodal analgesia, the low specificity suggests that this apparent agreement was largely driven by unidirectional recommendation patterns. Similarly, for regional block recommendations, high percentage agreement accompanied by low kappa values indicates that systematic recommendation behaviors may constrain statistical agreement, as previously described in the literature [11, 19, 20]. This phenomenon is consistent with the well-described prevalence effect, whereby near-uniform recommendations inflate observed agreement while attenuating kappa values.

Taken together, these findings suggest that AI systems can demonstrate a degree of contextual awareness in postoperative analgesia planning; however, such awareness does not consistently translate into alignment with real-world clinical decision-making. AI systems appear to exhibit more consistent decision patterns for salient clinical interventions, such as regional anesthesia, while struggling to adequately capture the heterogeneity of clinical practice in more nuanced domains, including opioid use and multimodal analgesia strategies [24, 25].

One of the principal strengths of this study lies in the evaluation of AI-generated recommendations against real-world patient data and analgesia strategies that were actually implemented in clinical practice. This approach enables assessment of the relationship between AI outputs and real clinical decision-making, rather than focusing solely on theoretical accuracy. Additionally, the use of advanced statistical techniques, including multivariable modeling and penalized logistic regression, enhances the methodological robustness of the findings.

From a practical clinical perspective, these findings indicate that large language model–based AI systems can meaningfully incorporate certain key perioperative contextual elements, particularly the presence of regional anesthesia, when generating postoperative analgesia recommendations. The relatively high concordance observed in regional block–related recommendations suggests that AI systems may provide clinically relevant support in specific domains of postoperative pain planning.

However, the more limited alignment observed in opioid-related decisions underscores that AI-generated recommendations should not be interpreted as standalone directives. Instead, they should be viewed as complementary decision-support tools that require integration with patient-specific factors, surgical context, and clinician judgment. When interpreted within this framework, AI systems may serve as useful adjuncts in postoperative analgesia planning, supporting—but not replacing—clinician-led decision-making.

### Limitations

Several regression estimates were characterized by wide confidence intervals or extreme odds ratios, reflecting sparse data and complete separation in certain models. These findings indicate limited statistical precision and should be interpreted with caution; accordingly, the present analyses emphasize the directionality and consistency of observed patterns across AI systems rather than the magnitude of individual effect estimates. This limitation is inherent to exploratory analyses of complex decision-support behaviors and highlights the need for larger datasets in future confirmatory studies.

This single-center study may have limited generalizability to other patient populations and clinical settings. In addition, AI systems were evaluated within a specific time frame using a standardized prompt; alternative model versions, system updates, or prompt structures could yield different outputs. Although expert evaluations were performed by experienced anesthesiologists, interindividual variability in clinical judgment may have influenced appropriateness ratings.

Finally, excluding patients with significant perioperative complications or postoperative ICU admission may limit applicability to lower-risk elective surgical populations. The higher prevalence of open surgery in the regional block group likely reflects confounding by indication. Although surgical approach was adjusted for in all models, residual confounding cannot be fully excluded due to the observational design.

## Conclusions

This study evaluated, using real-world patient data, the impact of regional anesthesia on postoperative analgesia recommendations generated by large language model–based artificial intelligence systems in patients undergoing elective abdominal surgery. The findings demonstrate that AI systems are capable of responding to the presence of regional blocks; however, the extent and clinical implications of this responsiveness vary across analgesic domains. While regional block presence markedly reduced the likelihood of additional regional anesthesia recommendations, it did not independently influence opioid-related recommendations. Moreover, multimodal analgesia was presented as a largely universal strategy by certain AI systems, with limited differentiation according to patient- or procedure-specific factors.

Expert evaluations indicated that AI-generated recommendations were perceived as more clinically appropriate when regional anesthesia information was incorporated into the clinical context. Nevertheless, agreement analyses revealed limited concordance between AI recommendations and real-world clinical practice, particularly with respect to opioid use and regional block decision-making. These findings suggest that, although AI systems may exhibit contextual awareness, this awareness does not consistently translate into alignment with the complexity and variability of actual clinical decision-making.

Overall, while AI-based systems hold potential as supportive tools in postoperative pain management, the present results do not support their use as autonomous decision-makers. The integration of artificial intelligence into perioperative analgesia planning should remain transparent, context-aware, and firmly guided by clinician oversight to ensure patient safety and clinical appropriateness.

## Supplementary Information

Supplementary Material 1.

## Data Availability

Additional data available from the authors upon specific request.

## Appendix 1. Complete prompt template used for AI queries

Prompt template used for all AI systems (ChatGPT, Gemini, Copilot):

You are an anesthesiology consultant.

Based on the following real-world patient and surgical information, generate a postoperative analgesia recommendation.

Please consider:

- Opioid use (yes/no and type if applicable)
- Multimodal analgesia
- The role of regional anesthesia in postoperative pain management

Do not provide explanations or references.

Provide a concise and clinically appropriate recommendation.

Patient characteristics:

- Age: [years]
- Sex: [male/female]
- Body mass index (BMI): [kg/m²]
- ASA physical status: [I–IV]

Surgical details:

- Type of abdominal surgery: [procedure name]
- Surgical approach: [open / laparoscopic]

Anesthesia details:

- Type of anesthesia: [general anesthesia ± regional anesthesia]
- Regional block performed: [none / TAP block / bilateral TAP block / quadratus lumborum block / erector spinae plane block]

Based on the above information, provide a postoperative pain management plan.

## Abbreviations

AI: Artificial intelligence
ASA: American Society of Anesthesiologists
BMI: Body mass index
LLM: Large language model
TAP: Transversus abdominis plane
