# Supplementary material for: The impact of regional block presence on large language model–based postoperative analgesia recommendations in abdominal surgery: a comparative study using real-world patient data
Source: BMC Anesthesiol. 2026 Apr 10;26:316. doi: 10.1186/s12871-026-03814-y (PMC13188664; doi:10.1186/s12871-026-03814-y)
Supplement: Supplementary file 1 — Supplementary Material 1. [file 12871_2026_3814_MOESM1_ESM.docx]

**The Impact of Regional Block Presence on Large Language Model–Based Postoperative Analgesia Recommendations in Abdominal Surgery: A Comparative Study Using Real-World Patient Data**

**Bahar USLU BAYHAN^1^_,_ Tuğçe GAZİOĞLU KİŞİ**(**Corresponding author)^1^**

**1.** Bahar USLU BAYHAN^1^ (specialist doctor),

^1^Gaziantep City Hospital, Department of Anesthesiology and Reanimation, Gaziantep, Turkey

E-mail(s) : [dr.b.uslu@gmail.com](mailto:dr.b.uslu@gmail.com)

Phone : +90 531 654 21 01

ORCID ID: 0009-0009-3052-2604

**2**. Tuğçe GAZİOĞLU KİŞİ^1^(specialist doctor), (**Corresponding author)**

^1^Gaziantep City Hospital, Department of Anesthesiology and Reanimation, Gaziantep, Turkey

E-mail: [tugce.gazioglu@inonu.edu.tr](mailto:tugce.gazioglu@inonu.edu.tr)

Phone: +90-506 9008822

ORCID ID: 0009-0002-2336-1848

**Article Type:** Original Article

**Conflict of interest**: There is no conflict of interest between the authors.

**Funding**: No financial support was received.

**Ethical Approval:** Gaziantep City Hospital Non-Interventional Clinical Research Ethics Committee (approval decision no: 312/2025)

**Clinical trial registration number:** : Not applicable

**Informed Consent:** Written informed consent was obtained from each patient following a detailed explanation of the study objectives and protocol.
